# A Prospective Study on the Efficacy of Two Different Phlebotonic Therapies as a Bridge to Surgery in Patients with Advanced Hemorrhoidal Disease

**DOI:** 10.3390/jcm10081549

**Published:** 2021-04-07

**Authors:** Raffaele Orefice, Francesco Litta, Angelo Parello, Veronica De Simone, Paola Campennì, Angelo Alessandro Marra, Carlo Ratto

**Affiliations:** 1 Proctology Unit, Fondazione Policlinico Universitario “A. Gemelli” IRCCS, Largo A. Gemelli, 8, 00168 Rome, Italy; r.orefice89@gmail.com (R.O.); francescolitta83@yahoo.it (F.L.); veronicadesimone@libero.it (V.D.S.); campennip@hotmail.it (P.C.); angeloalessandromarr@libero.it (A.A.M.); carloratto@tiscali.it (C.R.); 2 Department of Medicine and Translational Surgery, Università Cattolica del Sacro Cuore, Largo A. Gemelli, 8, 00168 Rome, Italy

**Keywords:** phlebotonic therapies, hemorrhoidal disease, surgical treatment, quality of life

## Abstract

The aims of this study were to evaluate the efficacy of two different phlebotonic therapies, preoperatively administered in advanced hemorrhoidal disease (HD) patients with recommendation for surgery, and to assess patient satisfaction after treatment. In this prospective observational study, 100 patients were preoperatively treated either with micronized purified flavonoid fraction (group A) or sublingual nano-emulsion flavonoid (group B). HD symptoms, local inflammation signs and patients’ satisfaction were evaluated at baseline visit (T0), after 4 weeks of therapy (T1) and 8 weeks after its discontinuation (T2). In group A, a significant improvement for all HD symptoms and inflammation signs was observed after therapy (T1), followed by a reduction of efficacy in T2, except for itching and edema. In group B, therapy had a significant benefit on symptoms and local inflammation at T1, which persisted after its discontinuation for all symptoms, and edematous hemorrhoids. In both groups, the satisfaction rate was “good” in 60% of patients and patients were statistically significant more satisfied at T1 compared with T2 (*p* = 0.0001). No adverse events were recorded. Preoperative treatment was safe and useful to optimize patients’ clinical condition prior to surgery.

## 1. Introduction

Hemorrhoidal disease (HD) is the most frequent benign proctologic condition, which affects a large portion of the world population [1], with greater incidences in higher socioeconomic status, middle age (45–65 years) [2,3] and during pregnancy and postpartum [4], and with a prevalence ranging between 4.4% in the general population and 36.4% in general practice [5].

Different symptoms can characterize HD, such as bleeding, prolapse, discomfort, itching, sometimes pain and, more rarely, soiling with hygiene problems.

Conservative approaches are indicated for low-grade HD, which are based on dietary and lifestyle recommendations [6]. Moreover, a pharmacological therapy with phlebotonics or vasoactive medications is indicated to prevent, relieve, minimize or control acute HD symptoms [7,8,9]. Avoiding or postponing invasive procedures of acute HD could be convenient to plan the most appropriate therapeutic approach [10].

Many different medications are available to treat and control HD, including analgesics and corticosteroid creams with a local action, or flavonoids and mesoglycan with a systemic action. The latter ones, also called phlebotonics, which act on the capillary and venous complex, are heterogeneous and widely investigated categories of drugs extracted from plants, initially used to treat chronic venous insufficiency and edema [11].

The role of flavonoids is still debated; however, they have an anti-inflammatory action in addition to affecting the venous tone, capillary permeability and lymphatic drainage [12,13,14,15].

Recent studies show that flavonoids represent a valid therapeutic alternative both in acute symptomatic HD and in “long-term” hemorrhoid treatment, to bridge patients to surgical treatment in a better condition [16,17,18]. In addition, a potential benefit has been observed in reducing post-hemorrhoidectomy symptoms [19,20]. However, the role of flavonoids to improve the clinical condition of patients with III- and IV-degree hemorrhoids (according to the Goligher classification [21]) awaiting surgery has not been fully understood.

Primary aim of this study was to evaluate the effects of two different phlebotonic preparations based on symptoms reported by patients and local inflammation sign caused by III- and IV-degree HD. The secondary aim was to evaluate patient satisfaction.

## 2. Materials and Methods

### 2.1. Study Setting and Approval

This was a prospective, monocentric observational study. The study was conducted between December 2018 and December 2019 and promoted by the Proctology Unit of the Fondazione Policlinico Universitario A. Gemelli IRCCS of Rome, Italy. The local Ethics Committee approved the study (Protocol n°1268–1301). All patients signed a written informed consent.

### 2.2. Inclusion and Exclusion Criteria

Patients were evaluated according to common clinical practice at our center: assessment of the medical history, including that specifically related to HD (physical examination performed by digital rectal examination and anoscopy). Moreover, all patients received hygienic-behavioral suggestions, and a diet rich in fiber and water was prescribed.

Inclusion criteria were as follows: age between 18 and 70 years, advanced HD (III and IV-degree hemorrhoids according to the Goligher classification [21]) with indication for surgery, consent to participate in the study and consent to attend all scheduled FU visits. Exclusion criteria were as follows: colorectal or anal cancer; obstructive defecation syndrome; irritable bowel syndrome; inflammatory bowel disease; coagulation disorders; other proctologic diseases, such as anal abscess or fistula; anal fissure or acute hemorrhoidal thrombosis; pregnancy; and anticoagulant or anti-aggregant intake for another disease.

### 2.3. Data Collection and Treatment

The overall study period was 12 weeks, which was divided as follows: baseline visit (T0), first follow-up visit after 4 weeks of therapy (T1), second follow-up visit 8 weeks after therapy discontinuation (T2) and preoperative.

During each visit, local signs of hemorrhoidal inflammation, such as mucosal dystrophy, dysepithelialization and edema, were assessed by digital rectal examination and anoscopy, and were classified by a 11-point Numeric Rating Scale (NRS—from 0 = perfect condition to 10 = the worst condition). In addition, at each visit, the most typical HD symptoms (e.g., bleeding, prolapse, itching/discomfort and anal pain) were assessed by means of a self-reported patient questionnaire, and each symptom was graded with a 10-point Visual Analogue Scale (VAS) scale (except for pain, which was evaluated with a 100-point VAS scale).

Patient satisfaction was evaluated at T1 and T2, using a 4-point Likert scale, ranging from 0 to 3 (0 = very dissatisfied, 1 = dissatisfied, 2 = satisfied, 3 = very satisfied). A score of 2 or 3 was considered as a “good” degree of satisfaction.

### 2.4. Phlebotonic Therapies: Composition and Mechanism of Action

Patients were treated by two different phlebotonic therapies. In group A, oral intake of two doses two times per day for 4 weeks of micronized purified flavonoid fraction with diosmin (450 mg) and hesperidin (50 mg) was administered. This medication has documented effects on the microcirculation and bleeding due to their antioxidant, anti-inflammatory and phlebotonic action [15,16,17]. In group B, oral intake of one sublingual sachet two times per day for 4 weeks of a nano-emulsion combining micronized diosmin (67.5 mg) with hesperidin (7.9 mg), boswellia (75 mg), lysine (37.5 mg), cysteine (21 mg), ruscus (25 mg), vitamin E (9 mg), copper (0.65 mg) and arginine (2.1 mg) was administered.

### 2.5. Statistical Analysis

Sample size calculation: In a pilot study conducted at our institution (unpublished data), we found that the mean values of the “bleeding score” assessed with the VAS scale before and after treatment with a flavonoids preparation were 6.5 and 4.5, respectively, with a standard deviation of 2; based on these data, and considering an alpha confidence level = 0.05 and a unilateral test, the number of patients needed to reach a 95% power was calculated to be 48 individuals for each group; after establishing a 5% drop-off rate, we determined that the total number needed for the present study was finally about 100 patients.

Continuous data were analyzed as mean (with SD) and compared, using the paired or unpaired samples *t*-test or Wilcoxon Mann-Whitney test or Friedman test. Categorical data were analyzed as frequencies and percentages and compared by using either chi-square or marginal homogeneity test, as necessary. A *p* < 0.05 was considered statistically significant. The analysis of variance (ANOVA) models for repeated measures were adopted to analyze the differences between more than 2 groups over the study period. All data recorded were collected with Excel spreadsheet and analyzed with SPSS statistical version 21.0 for Windows software (SPSS, Chicago, IL, USA).

## 3. Results

### 3.1. Descriptive Data

One hundred patients fulfilled the inclusion criteria: In group A, 50 patients were enrolled, 22 of whom were males, with a mean age of 46.9 ± 11.2 years, while group B consisted of 50 patients, 24 of whom were males, with a mean age of 47.0 ± 11.5 years. Patient baseline characteristics of the two groups are detailed in Table 1; they were similar, expect for dysepithelialization (group A 6.2 ± 2.4 vs. group B 5.3 ± 2.4 *p* = 0.045). No patient was lost to follow-up. No adverse events were registered in either group, due to the medication administered.

### 3.2. Group A: Effect of the Therapy

**Figure 1:**
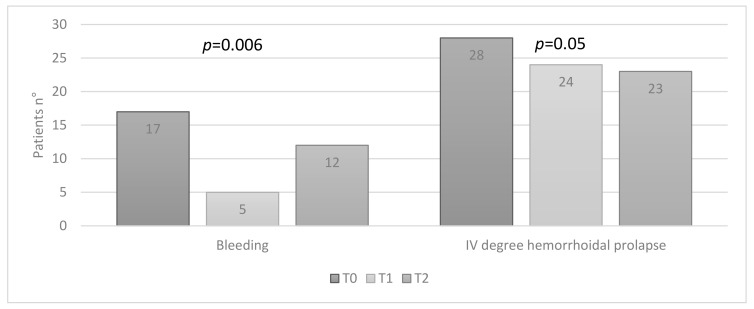
Group A: patients with bleeding and IV-degree hemorrhoidal prolapse at baseline visit (T0), first follow-up visit after 4 weeks of therapy (T1) and second follow-up visit 8 weeks after therapy discontinuation (T2) (Friedman test).

In group A, at T0, 17 (34%) patients had bleedings, and 28 (56%) IV-degree hemorrhoidal prolapse; at T1, 5 (10%) patients referred bleeding, and 24 (48%) IV-degree hemorrhoidal prolapse; at T2 visit, 12 (24%) patients had bleeding, and 23 (46%) IV-degree hemorrhoidal prolapse. A significant improvement in bleeding (*p* = 0.006) and a significant reduction in the degree of hemorrhoidal prolapse, from IV to III degrees (*p* = 0.050), were registered (Figure 1).

**Figure 2:**
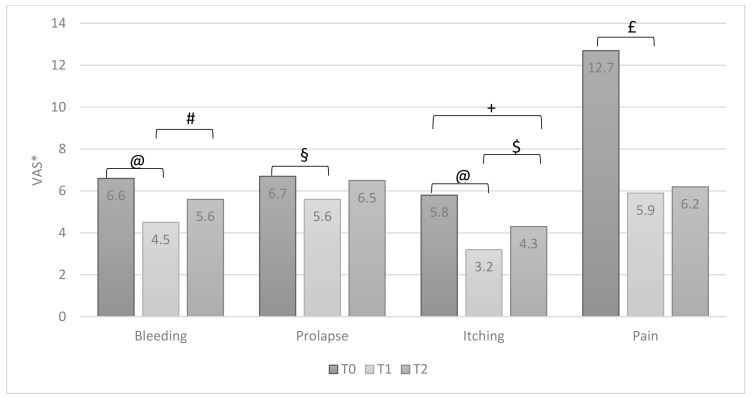
Group A: patients’ symptoms at T0, T1 and T2 (ANOVA test, values are means). * VAS: Visual Analogue Scale, at 10-points, except for pain, at 100-points. @ *p* = 0.0001; # *p* = 0.02; § *p* = 0.01; + *p* = 0.002; $ *p* = 0.042; £ *p* = 0.04.

**Figure 3:**
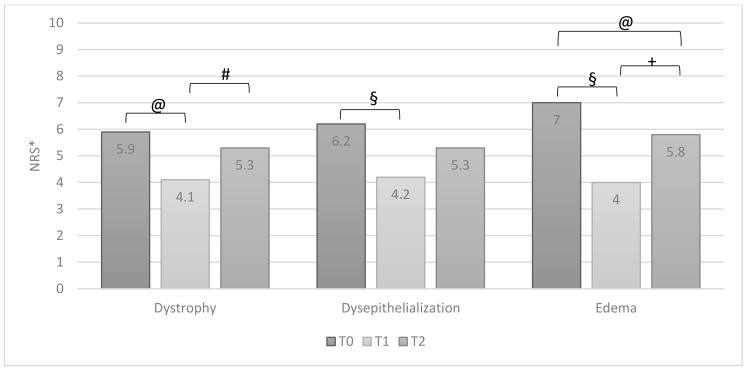
Group A: patients’ signs of inflammation T0, T1 and T2 (ANOVA test, values are means). * NRS: Numeric Rating Scale, 10-point scale. @ *p* = 0.001; # *p* = 0.04; § *p* = 0.0001; + *p* = 0.03.

All symptoms reported by patients significantly improved after therapy (T0 vs. T1) (Figure 2). Similarly, also, all signs of inflammation significantly improved (Figure 3). At the T2 visit, a significant reduction of therapeutic efficacy was observed for all symptoms and local signs of inflammation, except for itching (Figure 2) and edema (Figure 3). When comparing data of T1 with T2, a worsening of all domains was observed. It was statistically significant for bleeding and itching (Figure 2) and for dystrophy and edema (Figure 3).

### 3.3. Group B: Effect of the Therapy

**Figure 4:**
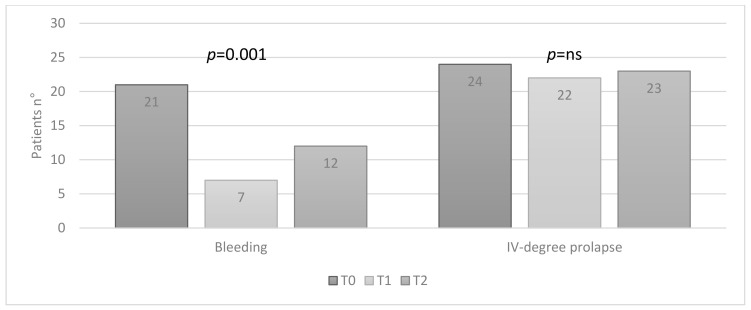
Group B: patients with bleeding and IV-degree hemorrhoidal prolapse, at T0, T1 and T2 (Friedman test).

In group B, at T0, 21 (42%) patients had bleeding and 24 (48%) IV-degree hemorrhoidal prolapse; at T1, 7 (14%) patients still had bleeding, and 22 (44%) IV-degree prolapse; at T2, 12 (24%) patients had bleeding and 23 (46%) IV-degree prolapse. A statistically significant improvement in bleeding was observed (*p* = 0.001), while no statistically significant reduction in the degree of prolapse was found (Figure 4).

**Figure 5:**
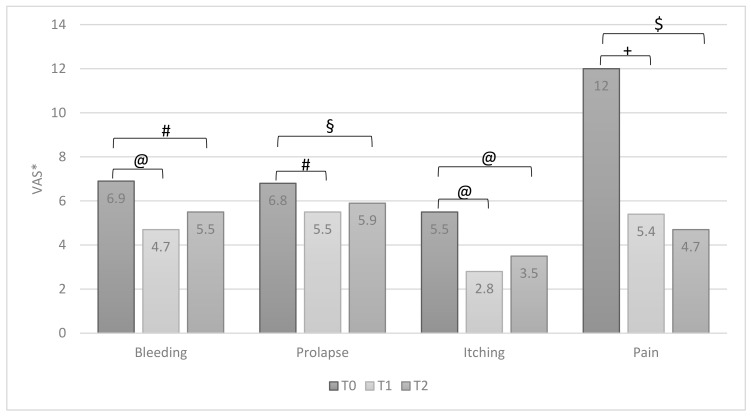
Group B: patients’ symptoms at T0, T1 and T2 (ANOVA test, values are means). * VAS: Visual Analogue Scale, at 10-points, except for pain, at 100-points. @ *p* = 0.0001; # *p* = 0.001; § *p* = 0.023; + *p* = 0.03; $ *p* = 0.01.

**Figure 6:**
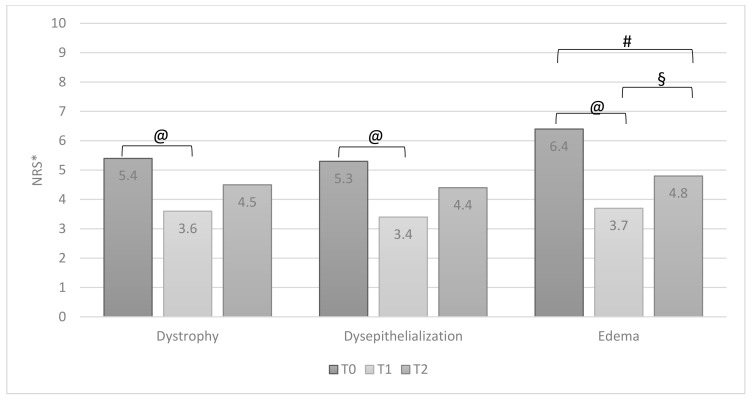
Group B: patients’ signs of inflammation T0, T1 and T2 (ANOVA test, values are means). * NRS: Numeric Rating Scale, 10-point scale. @ *p* = 0.0001; # *p* = 0.001; § *p* = 0.023.

For group B, data analysis showed a statistically significant improvement after therapy (T0 vs. T1) for all symptoms reported by patients (Figure 5) and for all signs of inflammation (Figure 6). Unlike group A, the efficacy of the therapy was still evident for all symptoms referred by patients (Figure 5) and edema (Figure 6), even after the discontinuation of therapy (T2). When comparing T1 and T2, the discontinuation of therapy showed a reduction in beneficial effects for all domains analyzed with a statistically significant worsening only for edema (Figure 6).

### 3.4. Patients’ Satisfaction

**Figure 7:**
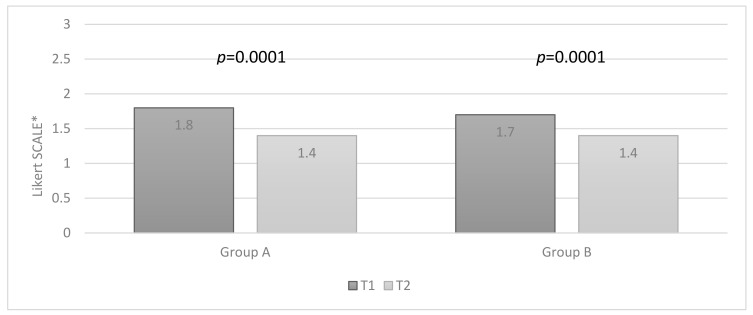
Patients’ satisfaction rate in both groups, comparing T1 and T2 visits (Wilcoxon test). * Likert Scale: four-point scale (0 to 3).

In both groups, the satisfaction score was “good” in 30 out of 50 (60%) patients, and patients were statistically significant more satisfied at T1, compared with T2 (mean value in group A, 1.8 ± 1.0 vs. 1.4 ± 0.9, *p* = 0.0001; in group B, 1.7 ± 1.0 vs. 1.4 ± 1.0, *p* = 0.0001) (Figure 7).

When comparing group A to group B, no statistically significant difference emerged regarding all clinical outcomes examined.

## 4. Discussion

The efficacy of phlebotonic therapy in HD treatment, widely studied and used in chronic vascular insufficiency, remains, among scientists and physicians, debatable [9,22]. This approach is usually indicated for low-grade HD [23,24]. Although the mechanism of action is not completely understood, several studies have demonstrated the efficacy of flavonoids in patients affected by HD. In 2006, Alonso-Coello et al. [16] conducted a meta-analysis, comparing the use of flavonoids versus placebo or no treatment in HD: 14 randomized trials were included, with 1514 participants, demonstrating a statistically significant improvement in bleeding, pain and itching using flavonoids. Other studies reported a significant benefit from using flavonoid preparations, particularly for bleeding [18,19,20,25]. Unfortunately, only a few studies evaluated the non-operative treatment with phlebotonic preparations in patients with advanced HD, confirming their usefulness and safety [26].

This study confirms and supports the beneficial effects of flavonoid-based therapy in patients affected by advanced HD candidate for surgery: Significant benefits on both symptoms and local inflammation signs were documented. In particular, we observed, at the end of the pharmacological treatment (T1), but also, in some regards, after up to an 8-week washout period from drug intake (T2), improvements. Reduction of treatment efficacy was observed especially for group A, when comparing the outcome at T1 and T2; however, also group B showed, at T2, a statistically significant improvement for all symptoms, including edema due to flavonoid therapy. This could be hypothetically attributed to the combination of flavonoids with other components (hesperidin, boswellia, lysine, cysteine, ruscus, vitamin E, copper and arginine). Therefore, both types of flavonoid-based therapies could be useful to control the disease in patients waiting for surgery, thus optimizing the hemorrhoidal condition for surgery.

More detailed, both medication regimens produced a significant benefit on hemorrhoids hemodynamic (reducing bleeding) and inflammation (reducing itching and pain and specific signs), but they did not impact significantly the hemorrhoidal prolapse, which was expected; in fact, no pharmacological treatment is nowadays able to significantly reduce and/or to restore the degradation of the extracellular matrix. This is the reason why, in the vast majority of patients complaining about hemorrhoidal prolapse, surgery represents a pivotal treatment. This gives a more significant value to patient satisfaction observed in this study, in which both medication regimens resulted as being “good” for the majority of patients with an advanced HD who required surgery anyway.

Limitations of this study are both the relatively small sample size and no evaluation of patients’ quality of life. Increasing the sample of the study could give even more solidity to the results obtained, highlighting the importance of preoperative treatment in patients with advanced disease, and this could be guaranteed by a multicenter study. We also believe that the quality of life of patients can be both a very useful element to evaluate patients with HD but also a useful tool to evaluate the benefit of a conservative or surgical treatment.

## 5. Conclusions

This study confirms that flavonoids and preparations including flavonoids with phlebotonic action could play a crucial role to treat patients with advanced HD. Their adoption was safe and well tolerated by patients in this study. Although, flavonoids usually cannot replace surgery in most advanced HD degrees, both therapies represented a valid “bridge-therapy” for patients waiting for surgery. They improved the hemorrhoidal condition during the overall study period (particularly for patients treated with sublingual nano-emulsion flavonoids), reducing severity and symptom occurrence, as well as local inflammation.

## Figures and Tables

**Table 1:** Patients’ baseline characteristics.

	Group A	Group B
Patients (n°)	50	50
Sex (M:F)	22:28	24:26
Age (years)	46.9 ± 11.2	47.0 ± 11.5
Comorbidities (n°)	20	24
Digital Rectal Examination		
- Bleeding (n°) - IV-degree prolapse (n°) - Dystrophy (NRS *) - De-epithelialization (NRS) - Edema (NRS)	17 28 5.9 ± 2.5 6.2 ± 2.4 7.0 ± 1.9	21 24 5.4 ± 2.3 5.3 ± 2.4 6.4 ± 2.0
Patients’ symptoms		
- Bleeding (VAS **) - Prolapse (VAS) - Itching (VAS) - Pain (VAS)	6.6 ± 2.0 6.7 ± 1.7 5.8 ± 2.2 12.7 ± 17.3	6.9 ± 1.7 6.8 ± 1.6 5.5 ± 2.2 12.0 ± 17.2

* NRS: Numeric Rating Scale, 11-point. ** VAS: Visual Analogue Scale, at 10-points, except for pain, at 100-points.

## Data Availability

The data presented in this study are available on request from the corresponding author upon reasonable request.
